# Precision Engineering of Chondrocyte Microenvironments: Investigating the Optimal Reaction Conditions for Type B Gelatin Methacrylate Hydrogel Matrix for TC28a2 Cells

**DOI:** 10.3390/jfb15030077

**Published:** 2024-03-20

**Authors:** Qichan Hu, Marc A. Torres, Hongjun Pan, Steven L. Williams, Melanie Ecker

**Affiliations:** 1 Department of Biomedical Engineering, University of North Texas, Denton, TX 76203, USA; 2 Department of Chemistry, University of North Texas, Denton, TX 76203, USA; 3 Department of Biological Sciences, University of North Texas, Denton, TX 76203, USA

**Keywords:** GelMA synthesis, hydrogel, mechanical and physical properties, 3D cell culture, cell viability, TC28a2 cells

## Abstract

Gelatin methacrylate (GelMA) is a photocrosslinkable biomaterial that has gained widespread use in tissue engineering due to its favorable biological attributes and customizable physical and mechanical traits. While GelMA is compatible with various cell types, distinct cellular responses are observed within GelMA hydrogels. As such, tailoring hydrogels for specific applications has become imperative. Thus, our objective was to develop GelMA hydrogels tailored to enhance cell viability specifically for TC28a2 chondrocytes in a three-dimensional (3D) cell culture setting. We investigated GelMA synthesis using PBS and 0.25M CB buffer, analyzed the mechanical and physical traits of GelMA hydrogels, and evaluated how varying GelMA crosslinking conditions (GelMA concentration, photoinitiator concentration, and UV exposure time) affected the viability of TC28a2 chondrocytes. The results revealed that GelMA synthesis using 0.25M CB buffer led to a greater degree of methacrylation compared to PBS buffer, and the LAP photoinitiator demonstrated superior efficacy for GelMA gelation compared to Irgacure 2959. Additionally, the stiffness, porosity, and swelling degree of GelMA hydrogels were predominantly affected by GelMA concentration, while cell viability was impacted by all crosslinking conditions, decreasing notably with increasing GelMA concentration, photoinitiator concentration, and UV exposure time. This study facilitated the optimization of crosslinking conditions to enhance cell viability within GelMA hydrogels, a critical aspect for diverse biomedical applications.

## 1. Introduction

Hydrogels represent a three-dimensional (3D) network of polymeric materials with hydrophilic functionality capable of retaining substantial amounts of water. These materials can be categorized based on their source and composition into natural, synthetic, and seminatural hydrogels [1]. Gelatin, derived from the partial hydrolysis of native collagen found in tissues such as skin, tendons, ligaments, or bones of animals such as cows or pigs, serves as a natural polymer for hydrogel fabrication. Depending on the method of processing, gelatin exists in two primary forms: type A (resulting from acid hydrolysis) and type B (resulting from alkaline hydrolysis) [2]. Gelatin exists as polypeptide chains, held together by hydrogen bonds between adjacent chains of amino acids, primarily glycine, proline, hydroxyproline, glutamine, arginine, alanine, asparagine, and others [3]. However, notable differences exist in amino acid composition across gelatin sources [4].

As a natural protein-derived polymer, gelatin finds extensive applications in the formulation of medical hydrogels owing to its favorable biocompatibility, biodegradability, low antigenicity, and inclusion of the bioactive motif arginine–glycine–aspartic acid (RGD) for facilitating cell adhesion and growth [5]. Moreover, compared with collagen, gelatin exhibits reduced immunogenicity, which can be attributed to the disruption of triple helix structures and degradation properties [6]. Nonetheless, its utility in biomedicine is constrained by inherent limitations such as low stability and inadequate mechanical strength [7]. Natural gelatin gelation relies solely on physical intermolecular interactions among its constituent polypeptide chains, rendering the resultant gels unstable at physiological temperatures and impeding the modification of their properties [8]. To address these shortcomings, chemical strategies have been employed to enhance hydrogel stiffness by incorporating organic reagents such as methacrylic anhydride, tannic acid, 1,6-diisocyanatohexane, and furfuryl amine [9,10,11,12]. Among these, methacrylation has predominated, pioneered by Van Den Bulcke et al. in 2000, involving the introduction of methacrylate groups into gelatin via reaction with activated acrylic acid and primary amines of lysine side chains [9]. The resulting GelMA macromers can be covalently crosslinked with ultraviolet (UV) light in a facile and controlled manner.

The synthesis of GelMA involves controlling various parameters to tailor its properties for specific applications in tissue engineering and regenerative medicine. These parameters include the selection of gelatin sourced from diverse origins, the ratio of methacrylic anhydride to gelatin, and reaction time. These factors collectively determine the extent of methacrylation of the gelatin molecules, directly impacting the degree of substitution and ultimately shaping the properties of the resulting GelMA. Additionally, reaction temperature and pH play significant roles in controlling reaction kinetics and the ionization state of functional groups, thereby affecting the efficiency of the methacrylation process. Moreover, the crosslinking conditions for GelMA to form hydrogels, including UV exposure time, GelMA concentration, and photoinitiator (PI) concentration, further impact the mechanical traits and biocompatibility of the resultant hydrogel. By precisely managing these parameters, researchers can tailor GelMA to meet the specific requirements of diverse biomedical applications.

GelMA is widely utilized in biomedical engineering owing to its advantageous characteristics, such as biocompatibility, customizable mechanical attributes, and capacity to facilitate cell adhesion and proliferation. The application of GelMA in 3D cell culture encompasses various aspects, such as scaffold fabrication, cell encapsulation, drug delivery, bioprinting, and organ-on-a-chip systems [13,14,15,16,17]. These applications leverage GelMA’s ability to provide a supportive environment for cells to grow and interact in 3D matrices, mimicking the in vivo microenvironment more closely than traditional 2D culture systems.

Chondrocytes have been extensively used in tissue engineering for cartilage regeneration and repair. They are the primary cell type found in cartilage and are responsible for synthesizing and maintaining the extracellular matrix (ECM) of the tissue [18]. However, investigations involving human chondrocytes have encountered obstacles in obtaining ample quantities of primary cells from a single joint and inherent variabilities among donors, including age and medical background. As a result, the exploration of immortalized cell lines has emerged as a promising avenue to address these limitations. TC28a2 is a chondrocyte cell line derived from human articular cartilage, making it physiologically relevant for studying human cartilage biology. Notably, TC28a2 cells exhibit chondrogenic characteristics, including the expression of cartilage-specific markers such as type II collagen and aggrecan. When encapsulated in a hydrogel matrix, these cells can maintain their chondrogenic phenotype and contribute to the formation of cartilage-like tissue [19]. Moreover, their proliferative capacity in vitro allows researchers to generate enough cells for tissue engineering applications with a consistent platform for their experiments. Furthermore, TC28a2 cells exhibit consistent behavior and reproducible results, making them a reliable choice for cartilage tissue engineering studies. Researchers can expect uniform cell behavior and matrix production, which are essential for controlling the properties of the engineered cartilage tissue.

Various types of hydrogels have been used for chondrocyte culture and tissue engineering applications due to their biocompatibility, tunable properties, and ability to mimic the native extracellular matrix (ECM) of cartilage. Some commonly used hydrogels for chondrocyte culture include agarose [20], alginate [21], hyaluronic acid [22], collagen [23], chitosan [24], fibrin [25], GelMA [26], and poly(ethylene glycol) (PEG) [27]. Each type of hydrogel has its own prominent advantages and disadvantages. Among these hydrogels, collagen and fibrin and GelMA hydrogels facilitate direct cell adhesion to ligands on these proteins, whereas other hydrogels, such as agarose, alginate, hyaluronic acid, and PEG, lack specific binding sites, thereby impeding direct cell adhesion [28,29,30]. Collagen, chitosan, fibrin, and PEG hydrogels are often engineered to have a higher mechanical strength compared to agarose, alginate, hyaluronic acid, and GelMA hydrogels [31,32,33]. There is not a universal standard for choosing a hydrogel for a particular application, as the selection process depends on various factors specific to the intended use. We chose GelMA because of its dual advantages: excellent cell attachment properties and tunable mechanical properties. GelMA, derived from gelatin, possesses inherent cell-binding motifs that promote cell adhesion, proliferation, and differentiation [5]. Additionally, its mechanical properties can be controlled through crosslinking parameters such as UV duration, photoinitiators, and GelMA concentration [34]. This unique combination of natural cell affinity and tunable mechanical characteristics makes GelMA an ideal choice for our research in tissue engineering and regenerative medicine.

While GelMA has been widely explored for its utility in 3D cell culture and tissue engineering, extensive literature reviews reveal great heterogeneity in its synthesis process and resultant physiomechanical characteristics. Thus far, a universally accepted standard for GelMA synthesis has not been established. Moreover, there is often a lack of systematic guidance on customizing GelMA for specific cell types and intended applications. This study aimed to clarify the GelMA synthesis process, determine the optimal reaction conditions, and elucidate how diverse polymerization parameters impact the ultimate properties of hydrogels. Consequently, we will investigate how these properties influence cellular proliferation within such constructs.

## 2. Materials and Methods

### 2.1. Synthesis of GelMA

#### 2.1.1. Preparation of GelMA Precursor

**Figure 1:**
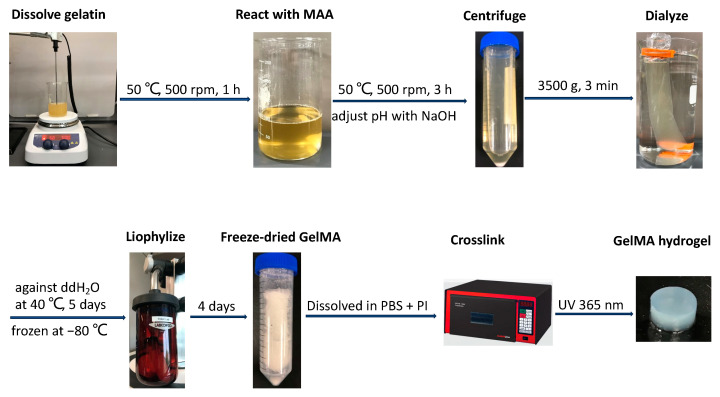
Schematic illustration of GelMA hydrogel synthesis.

GelMA was synthesized as previously described [35]. As illustrated in Figure 1, gelatin from bovine skin, type B (~225 g Bloom, Sigma-Aldrich, St. Louis, MO, USA), was fully dissolved in reaction buffer under magnetic stirring at 500 rpm for 1 h at 50 °C. Methacrylic anhydride (MAA, Sigma-Aldrich) was added to the 10% *w*/*v* gelatin solution in a drop-wise manner, and the reaction was carried out under constant stirring at 500 rpm to induce methacrylation for 3 h at 50 °C. The solution was then transferred to separate 50 mL conical tubes and centrifuged at 3500× *g* for 3 min to precipitate unreacted materials or impurities. The supernatant was collected in a beaker and diluted three times with prewarmed deionized water, and the resulting solution was dialyzed through a cellulose membrane (molecular weight cutoff: ≈12–14 kDa) against distilled water at 40 °C for 5 days with two water changes per day. After being stored at −80 °C overnight, the solution was freeze-dried in a lyophilizer (Labconco, Kansas, MO, USA) for 4 days to generate a porous white foam and stored at −80 °C until further use. To optimize the conditions of GelMA synthesis, phosphate-buffered saline (PBS, pH 7.4) and 0.25 M carbonate-bicarbonate buffer (CB, pH 9.0) were used as reaction buffers with/without pH adjustment, and various MAA (mL)/gelatin (g) feed ratios of 0.1:1, 0.2:1, 0.4:1, 0.6:1, 0.8:1, and 1:1 were also investigated.

#### 2.1.2. Degree of Substitution

To verify the methacrylation, proton nuclear magnetic resonance (1H NMR) spectroscopy was conducted with a Varian NMR spectrometer (500 MHz, Agilent Technologies, Santa Clara, CA, USA). The gelatin and GelMA samples were dissolved separately in deuterium oxide (D_2_O) at a concentration of 20 mg/mL. The NMR experiments were carried out using the standard one-pulse sequence, comprising 32 scans, a relaxation delay of 1 s, and a 45-degree excitation pulse duration of 2.85 μs. All the spectra were measured at room temperature. The chemical shifts are presented in parts per million (ppm).

The degree of substitution (DOS) was measured with TNBS (2,4,6-trinitrobenzenesulfonic acid, Sigma-Aldrich, St. Louis, MO, USA) by quantifying the remaining free amino groups in GelMA. This assay was performed according to the published method [36], with minor variations. Briefly, GelMA and type B gelatin were separately dissolved at 0.2 mg/mL in reaction buffer (0.1 M NaHCO_3_, pH 8.5). Then, 0.25 mL of 0.01% TNBS solution was added to 0.5 mL of each sample solution and incubated at 37 °C for 2 h. Finally, the reaction was stopped by adding 250 μL of 10% (*w*/*v*) SDS and 125 μL of 1 N HCl. A control group without gelatin or GelMA was included to correct for potential absorption from the solution itself. The absorbance of each sample was measured at 335 nm using a microplate reader (BioTek, Winooski, VT, USA). The following equation was then used to calculate the conversion of amine groups [37]:

(1) $$Conversation\left(\%\right)=[1-(\frac{Absorbance\;of\;GelMA-Absorbance\;of\;Control}{Absorbance\;of\;Gelatin-Absorbance\;of\;Control})]\times 100$$

#### 2.1.3. Photo-Crosslinking of GelMA Hydrogels

The GelMA macromers were dissolved in 1X PBS at concentrations of 5%, 10%, 15%, and 20% (*w*/*v*). For each solution, either lithium phenyl-2,4,6-trimethylbenzoylphosphinate (LAP; Sigma-Aldrich) or 2-hydroxy-4′-(2-hydroxyethoxy)-2-methylpropiophenone (Irgacure 2959; Sigma-Aldrich) was added at concentrations of 0.005%, 0.01%, 0.025%, 0.05%, 0.1%, 0.25%, and 0.5% (*w*/*v*), respectively. The mixtures were kept at 40 °C for 30 min to ensure the formation of uniform solutions. GelMA hydrogels were then fabricated by dispensing 200 µL of each reaction mixture into a 1.5 mL Eppendorf tube. Subsequently, the samples were subjected to 365 nm UV light exposure for various durations of photocuring (0.5, 1, 2, 4, 6, 8, and 10 min). The samples were positioned at the base of the chamber of a UVP crosslinker (CL-3000L, Analytik Jena, Jena, Germany) with a UV intensity of 100 μW/cm^2^. The gelation of GelMA was monitored by observation. GelMA samples were fully gelled if no free-flowing liquid was observed after the tube was vigorously shaken and inverted. Conversely, GelMA was considered nongelled if the solution remained entirely liquid. A state between these two conditions signaled incomplete GelMA gelation.

### 2.2. Characterization of GelMA Hydrogels

#### 2.2.1. Compressive Modulus

The stiffness of the GelMA hydrogels was assessed by determining the compressive modulus using a MicroTester instrument (CellScale, Waterloo, ON, Canada). The samples were prepared as 4 mm × 4 mm cylinders using a biopsy punch. Subsequently, they were immersed in PBS at room temperature for 24 h to achieve swelling equilibrium before testing. The experimental setup involved fixing a tungsten microbeam, 1.5748 mm in diameter, to a vertical actuator on one end and to a 5 × 5 mm^2^ compression platen on the other end. A camera was used to track the displacement of the compression platen. As the beam compressed the hydrogel, deflection was monitored in real-time by comparing the relative positions of the beam at the camera and at the motor. The gel was compressed to a final strain of 5% within 20 s, held for 5 s, and allowed to recover within 20 s. The compressive modulus was determined as the slope of the linear region of the stress–strain curve.

#### 2.2.2. Scanning Electron Microscopy

The morphological properties of the hydrogels were characterized by scanning electron microscopy (SEM). The hydrogels were first lyophilized and then sliced to expose their cross-sections. The inner structure of the samples was examined using an SEM TM3030Plus (Hitachi, Tokyo, Japan) with an operating voltage of 15 kV.

#### 2.2.3. Degree of Swelling

The degree of swelling of the GelMA hydrogels was determined by the amount of water absorbed by the freeze-dried samples. First, freshly prepared hydrogels were washed for 5–10 min to remove impurities. Then, the samples were freeze-dried in a lyophilizer for 24 h and weighed to determine the mass of the crosslinked hydrogels (weight of dry gel, W_d_). Following this, gels were rehydrated in PBS at room temperature for 48 h and re-weighed (weight of swollen gel, W_s_). The degree of swelling was calculated using the following equation [38]:

Degree of swelling (%) = [(W_s_ − W_d_)/W_d_] × 100

### 2.3. Effects of Photoinitiator and UV Exposure Time on Cell Viability in 2D Culture

#### 2.3.1. Cell Culture

The TC28a2 chondrocyte cell line was provided by Dr. Miguel Otero from the HSS Research Institute. These cell lines were established by Mary B. Goldring and derived as described in the literature [39,40,41]. The cells were cultured in DMEM: F-12 Medium (ATCC, Manassas, VA, USA) supplemented with 10% fetal bovine serum (Gibco, Thermo Fisher Scientific, Waltham, MA, USA) and 100 U/mL penicillin–streptomycin (Invitrogen, Thermo Fisher Scientific, Waltham, MA, USA) and incubated at 37 °C with 5% CO_2_. At 80–90% confluency, the cells were detached from the culture dishes using a 0.25% trypsin-EDTA solution, collected via centrifugation, and quantified using an automated cell counter (Countess II, Thermo Fisher Scientific, Waltham, MA, USA). Subsequently, the cells were resuspended in culture medium for subsequent passage and experimental procedures.

#### 2.3.2. MTT Assay

To evaluate the effects of photoinitiator concentration and UV exposure time on cell survival in 2D culture, an MTT assay was used to measure cellular metabolic activity as an indicator of cell viability. Initially, cells were seeded in 24-well plates at a density of 50,000 cells per well and allowed to adhere and proliferate for 24 h. Subsequently, the culture medium was replaced with fresh medium containing varying concentrations of the photoinitiator LAP (ranging from 0.01% to 0.5% *w*/*v*), and the plates were exposed to 365 nm UV light for specified durations (0, 1, 2, 4, 6, and 8 min) before being returned to the incubator. Following an additional 24 h and 48 h incubation periods, the medium was aspirated, and MTT solution (R&D systems, Minneapolis, MN, USA) was added to each well at a final concentration of 0.5 mg/mL. The cells were then incubated for 4 h, after which the medium was replaced with 500 μL of DMSO. Subsequently, 100 μL of the resulting solution from each well was transferred to a 96-well plate, and the absorbance was measured at 570 nm using a microplate reader. Cell viability was calculated as a percentage relative to the untreated control using the following equation:

Cell viability (%) = (A_treated_/A_control_) × 100 (where, A = absorbance) (2)

### 2.4. Cell Viability in 3D Culture

#### 2.4.1. Cell Encapsulation in GelMA Hydrogels

For 3D cell culture experiments, cells were mixed at a density of 1 × 10^7^ cells/mL with GelMA solution, which was prepared in PBS with photoinitiators and sterilized through 0.22 μM membrane filters prior to encapsulation. Then, hydrogel discs were formed by dropping 40 μL of the mixture in each well of 24-well plates and exposing them to 365 nm UV light for the required time. The cell-laden hydrogel discs were cultured in identical conditions as the 2D cell cultures, with a medium change every 2 days.

#### 2.4.2. Live/Dead Staining of Chondrocytes

Chondrocyte viability was determined by the LIVE/DEAD™ Viability/Cytotoxicity Kit (Invitrogen, Thermo Fisher Scientific, Waltham, MA, USA). Moreover, 24 h post-encapsulation, the cell-laden hydrogels were washed twice, 15 min per wash, with PBS. They were then stained with 2 μM calcein-acetoxymethyl (calcein-AM) and 4 μM ethidium homodimer-1 (EthD-1) in PBS, followed by incubation at 37 °C for 30 min. The live cells were stained green with calcein-AM, while the dead cells were stained red with EthD-1. The stained samples were washed with PBS again and imaged through a fluorescence microscope (Keyence, Osaka, Japan). The images were analyzed using ImageJ 1.54g (National Institutes of Health, Bethesda, MD, USA).

#### 2.4.3. AlamarBlue Assay

The alamarBlue assay™ was used to indirectly quantify the cell viability of chondrocytes. Briefly, after a 24-h encapsulation period, the culture medium was replaced with fresh medium containing 10% (*v*/*v*) alamarBlue (Thermo Fisher Scientific, Waltham, MA, USA), and the cells were incubated for an additional 24 h. Afterward, 100 μL of medium from each tested well was transferred to a 96-well plate, and the absorbance was read at wavelengths of 570 nm and 600 nm, respectively. The reduction of alamarBlue was calculated according to the manufacturer’s instructions.

#### 2.4.4. Hematoxylin and Eosin Staining

Hematoxylin and eosin (H&E) staining was conducted to directly observe the morphological changes of the cells within the hydrogels. Initially, the samples were washed with PBS and fixed overnight in 4% PFA. They were dehydrated using a series of ethanol solutions ranging from 70% to 100%, embedded in Paraplast^®^ (McCormick™ Scientific, Saint Lois, MO, USA), and sectioned to obtain cross-sections with a thickness of 7 μM. These cross-sections were then deparaffinized and subjected to staining with Harris hematoxylin and eosin dyes to visualize cell morphology. The detailed steps were listed in Supplementary Materials Tables S1 and S2. Finally, the stained samples were examined and imaged under an optical microscope (AmScope, Irvine, CA, USA).

### 2.5. Statistical Analysis

All the statistical analyses were performed using SPSS 27.0 software (IBM, Armonk, NY, USA), and all data were presented as the means ± standard errors. Statistical significance (* *p* < 0.05, ** *p* < 0.01) was determined using one-way ANOVA and repeated measures ANOVA. At least three independent experiments were performed for each condition in this study.

### 2.6. AI-Assisted Tools

The language was refined and professionalized using ChatGPT3.5 to enhance clarity and professionalism.

## 3. Results

### 3.1. GelMA Synthesis

**Figure 2:**
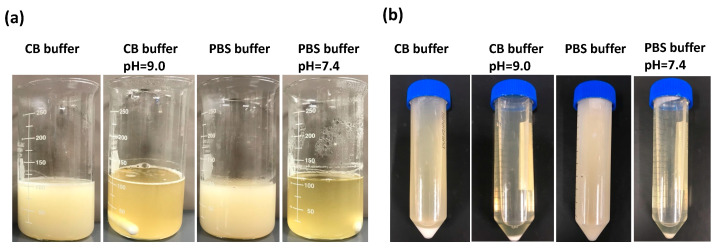
The appearance of reaction solutions during GelMA synthesis. (**a**) Appearance after a 3 h reaction of gelatin and methacrylic anhydride at 500 rpm at 50 °C. (**b**) Appearance after centrifugation of the reacted mixture at 3500 rpm for 3 min.

In this study, we initially adjusted the pH of the PBS buffer to 7.4 and the pH of the 0.25M CB buffer to 9.0 before investigating the impact of different pH values on the reaction between gelatin and MAA. Throughout the process, two groups were left without adjustment, while the others were maintained at their respective pH levels using 1 N NaOH. We monitored the pH changes in each reaction solution every 30 min for the duration of the synthesis, as outlined in Table 1. In the first hour, there was a significant decrease in pH for both PBS and 0.25M CB reaction solutions, whose pH was not adjusted. As such, the pH changes were marginal for the remaining duration. In addition, the mixture exhibited a white-milky appearance at the end of the 3-h reaction, and the white viscous substance was separated after centrifugation (Figure 2). For the two pH-adjusted groups, the reaction mixture appeared clearer, with fewer precipitates observed after centrifugation. This highlights the necessity for pH adjustment to ensure a thorough chemical reaction.

**Figure 3:**
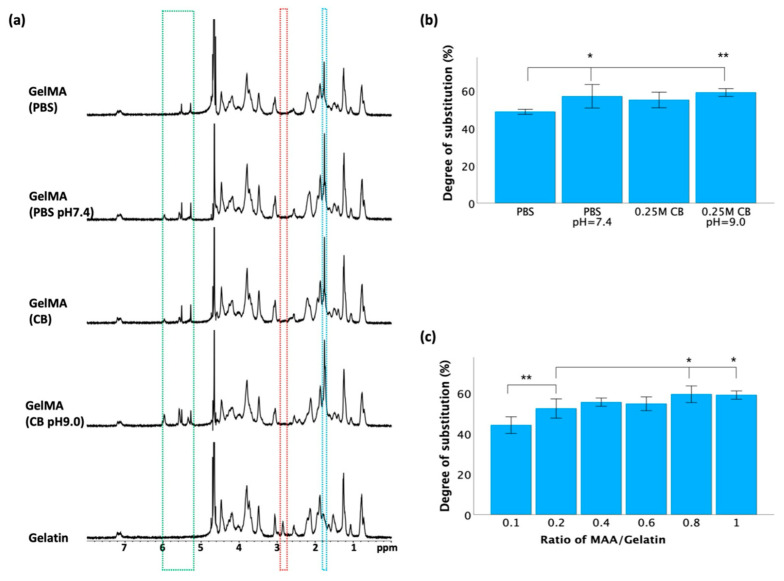
Verification of GelMA synthesis after a 3 h reaction. (**a**) 1H NMR spectra of GelMA and gelatin. (**b**) Degree of substitution assessed through TNBS assay at different pH conditions. (**c**) Degree of substitution assessed through TNBS assay at different ratios of MAA/gelatin. Data in (**b**,**c**) is presented as mean ± standard error, and statistical significance (* *p* < 0.05, ** *p* < 0.01) was determined using one-way ANOVA.

Nuclear magnetic resonance (NMR) spectroscopy was employed to validate the synthesis of GelMA from gelatin and MAA after a 3 h reaction. As depicted in Figure 3a, the spectra of the gelatin derivatives revealed distinct peaks compared to those of unmodified gelatin. In the HNMR spectra of the GelMA samples, more prominent peaks at approximately 1.8 ppm (blue frame) and 5.2–6.1 ppm (green frame) were observed, while the peak at approximately 2.9 ppm (red frame) vanished. The emergence of these new peaks in the HNMR spectra served as evidence of successful GelMA synthesis.

TNBS was used to determine the degree of substitution (DOS) after a 3 h reaction, which indicates the extent of methacrylation of gelatin molecules. As shown in Figure 3b, maintaining a constant pH led to a significant increase in the DOS compared to that in reactions conducted without pH adjustment, but there was no significant difference between the PBS and 0.25 M CB groups with pH adjustment. To further optimize the synthesis conditions, various batches of GelMA were synthesized using 0.25 M CB buffer (pH 9.0) by varying the ratio of methacrylic anhydride (MAA)/gelatin from 0.1 to 1 mL/g. As shown in Figure 3c, there was a significant difference between the 0.1:1 group and the other groups, yet no significant difference was observed when the ratio reached and exceeded 0.4.

### 3.2. Gelation Study

The photoinitiators Irgacure 2959 and LAP were evaluated for their ability to polymerize GelMA hydrogel precursors. The images of the time-course of the crosslinking process are displayed in Supplementary Figure S1, while the results are summarized in Table 2. The results indicated that LAP at 365 nm UV light irradiation was more effective than Irgacure 2959. For instance, practically none of the samples were crosslinked using 0.005% Irgacure 2959, while all the samples exhibited partial crosslinking when treated with 0.005% LAP. When employing a blend of 20% GelMA and subjecting it to 4 min of UV exposure, complete crosslinking was achieved with 0.01% LAP, whereas 0.5% Irgacure 2959 was necessary. Evidently, LAP exhibited a shorter duration of solution polymerization compared to Irgacure 2959 under 365 nm illumination at similar intensities and initiator concentrations. Additionally, the results presented in Table 2 demonstrated that the degree of hydrogel cross-linking was influenced by factors such as the GelMA concentration, duration of UV light exposure, and photoinitiator concentration. For example, when utilizing 0.01% LAP for 1 min, partial crosslinking was observed in the 5% and 10% GelMA solutions, whereas complete crosslinking was achieved in the 15% and 20% GelMA solutions. Comparatively, combining 20% GelMA with 0.01% Irgacure 2959 did not yet induce crosslinking within a 6 min UV exposure period. However, extending the UV exposure time to 8 min initiated partial crosslinking in the solution. After 2 min of UV exposure for GelMA solutions ranging from 5% to 20%, no crosslinking was observed across concentrations of Irgacure 2959 ranging from 0.005% to 0.25%. Nevertheless, crosslinking became evident in all GelMA solutions when the concentration of Irgacure 2959 was increased to 0.5%, displaying partial crosslinking after 2 min of UV exposure and complete crosslinking after a longer period of UV irradiation.

### 3.3. Mechanical and Physical Properties of GelMA Hydrogels

**Figure 4:**
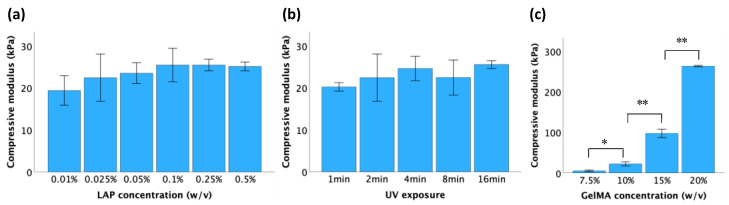
Compressive moduli of GelMA hydrogels when crosslinked at the following conditions: (**a**) 10% GelMA, UV exposure for 2 min; (**b**) 10% GelMA, LAP concentration of 0.025%; (**c**) UV exposure for 2 min, LAP concentration of 0.025%. All the data is presented as mean ± standard error, and statistical significance (* *p* < 0.05, ** *p* < 0.01) was determined using one-way ANOVA.

Unconfined compression tests were conducted on samples synthesized under varying conditions to determine the stiffness of GelMA. As shown in Figure 4a,b, within a certain range, there was no significant difference in the stiffness of the hydrogel of the same concentration (10%) as the LAP concentration and UV exposure time increased. However, a higher concentration of GelMA led to a significant increase in the GelMA hydrogel compressive modulus (Figure 4c). The compressive moduli of 7.5%, 10%, 15%, and 20% GelMA were 5 ± 1 kPa, 22 ± 3 kPa, 97 ± 5 kPa, and 263 ± 1 kPa, respectively. Thus, the mechanical properties of GelMA hydrogels can be effectively regulated by adjusting the concentration of GelMA.

**Figure 5:**
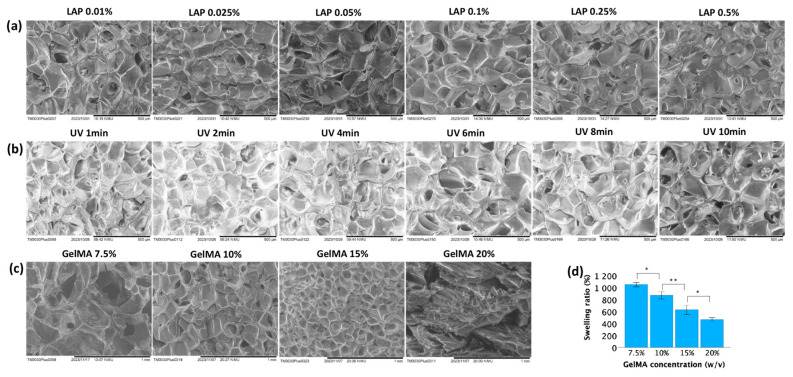
Physical properties of GelMA hydrogels. (**a**) SEM images of 10% GelMA subjected to 2 min of UV exposure with varying concentrations of LAP. (**b**) SEM images of 10% GelMA with 0.025% LAP under varying UV exposure times. (**c**) SEM images of GelMA hydrogels with different gel concentrations using 0.025% LAP and a UV exposure time of 2 min. (**d**) Swelling ratio of GelMA hydrogels with different gel concentrations using 0.025% LAP and a UV exposure time of 2 min. Data in (**d**) is presented as mean ± standard error, and statistical significance (* *p* < 0.05, ** *p* < 0.01) was determined using one-way ANOVA.

The morphological properties of the GelMA hydrogels were examined by scanning electron microscopy (SEM). As shown in Figure 5, microporous structures developed within the hydrogel during polymerization. These pocket-like pores were separated by the thin walls of the hydrogel matrix. The pore size decreased as the GelMA concentration increased (Figure 5c), while increasing the UV exposure time (at constant GelMA and LAP concentrations of 10% and 0.025%, respectively; Figure 5b) or LAP concentration (at 10% GelMA and 2 min UV exposure; Figure 5a) did not significantly affect the pore size.

Lower GelMA concentrations exhibited higher swelling ratios. Figure 5d showed that after immersion in PBS for 48 h, the mean swelling ratios for 7.5%, 10%, 15%, and 20% GelMA were 1047 ± 18%, 869 ± 30%, 626 ± 39%, and 465 ± 15%, respectively. These values demonstrated significant differences among each other.

### 3.4. Evaluation of the Cytotoxicity of LAP and UV Exposure

**Figure 6:**
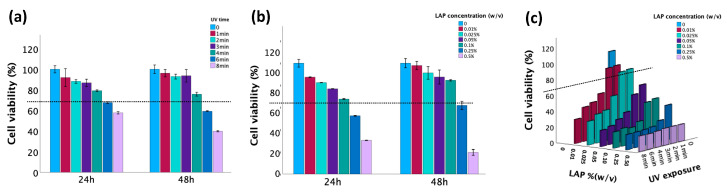
Cell viability measured by MTT. (**a**) Effect of photoinitiator LAP on cell viability assessed after 24 h and 48 h incubation, respectively. (**b**) Effect of UV exposure time on cell viability assessed after 24 h and 48 h incubation, respectively. (**c**) The combined effect of UV and LAP on cell viability assessed after 24 h of incubation. The dotted line represents 70% cell viability.

The cytotoxicity induced by LAP at different concentrations and UV exposures was assessed by the MTT assay. According to ISO 10993-5 [42], a material is considered biocompatible when the cell viability is ≥70% in in vitro testing when compared to untreated controls. As demonstrated in Figure 6, cell viability exceeded 70% when either the duration of UV exposure did not exceed 4 min or the LAP concentration did not exceed 0.1%. However, when these factors were combined, their synergistic effect led to a significant decrease in cell viability. Thus, cell viability values larger than 70% were obtained when the LAP concentration was ≤0.025% and the UV exposure time was ≤2 min.

### 3.5. Biological Analysis of Cell Behavior in GelMA Hydrogels

**Figure 7:**
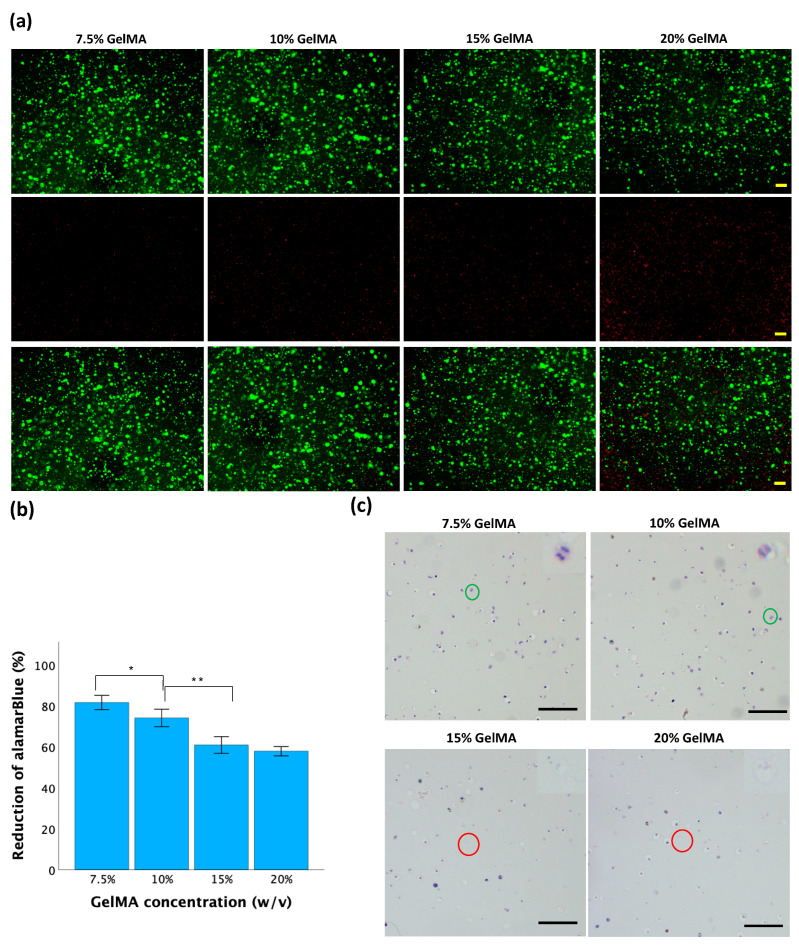
Cell behavior in GelMA hydrogels. (**a**) Cell viability by live/dead fluorescence staining. Live cells are green (top row), and dead cells are red (center row). The bottom row displays an overlay of the first and second rows. The scale bar is 200 μM. (**b**) Cell viability by alamarBlue assay. Data is presented as mean ± standard error, and statistical significance (* *p* < 0.05, ** *p* < 0.01) was determined using one-way ANOVA. (**c**) H&E staining of cell-laden GelMA hydrogels. Green circles indicate mitosis, and red circles indicate cellular fragmentation. The scale bar is 100 μM.

Cell viability was further tested with different concentrations of GelMA, 0.025% LAP, and 2 min of UV exposure. After 24 h of incubation, fluorescence staining was performed to estimate cell viability. As shown in Figure 7a, a predominance of green fluorescence suggests a high proportion of viable cells with intact membranes, indicating good cell viability. Conversely, an abundance of red fluorescence indicated more dead cells with compromised membrane integrity, suggesting reduced viability or cytotoxic effects. Elevated GelMA concentration led to an increase in red fluorescence signal intensity, indicating reduced cell viability.

The alamarBlue assay was used to measure metabolic activity, which serves as an indirect indicator of cell viability [43]. As shown in Figure 7b, the mean reductions of alamarBlue in the 7.5%, 10%, 15%, and 20% GelMA hydrogels were 81 ± 2%, 74 ± 2%, 61 ± 2%, and 58 ± 1%, respectively. As the GelMA concentration increased, the reduction of alamarBlue decreased. Significant differences were observed among all groups except for the comparison between the 15% GelMA and 20% GelMA groups.

Hematoxylin and eosin (H&E) staining was used to observe the cellular morphology and structure inside the hydrogel discs. As shown in Figure 7c, rounded cells were evenly distributed in the hydrogel matrix of all the samples. Within 7.5% and 10% GelMA hydrogels, there were more viable cells with well-defined cellular boundaries, intact nuclei, and observable mitotic division. Within 15% and 20% hydrogels, increased cellular shrinkage, nuclear condensation, or fragmentation was observed, indicating compromised cell viability or cellular stress within the hydrogel.

## 4. Discussion

GelMA is formed by the reaction between methacrylic anhydride (MAA) and gelatin. Through the reaction, a large number of amino groups on the side chains of gelatin are replaced with methacryloyl groups from MAA, introducing polymerizable methacrylamide groups to the gelatin macromers [34]. The main byproduct of GelMA synthesis is methacrylic acid, a carboxylic acid generated through the esterification of gelatin with methacrylic anhydride. As a carboxylic acid, methacrylic acid can release protons when dissolved in water, leading to an increase in the hydrogen ion concentration and a subsequent decrease in pH levels [28]. A significant decrease in pH can result in the protonation of the carboxyl groups present in gelatin, diminishing their reactivity toward the anhydride groups in methacrylic anhydride [36]. This phenomenon may decelerate or impede the esterification reaction, consequently lowering the GelMA yield and altering the properties of the resulting hydrogel. Thus, maintaining the pH within an appropriate range is crucial for effective GelMA synthesis. As shown in Figure 2, in the groups without pH adjustment, the mixture remained milky after the designated reaction time, which could suggest the presence of suspended particles or aggregates, indicating that the reaction may not be fully complete. However, with pH adjustment, a clearer mixture was formed, and less material was precipitated. At a pH range of approximately 9, the gelatin-based system surpasses the isoelectric point (IEP) of gelatin B, which is 4.7–5.6 [44]. At this pH value, the carboxyl groups (-COOH) of the aspartic and glutamic acid residues within the polypeptide chains of gelatin exist in their carboxylate form (-COO-). These unprotonated carboxylate groups contribute to the high water solubility of gelatin under the conditions required for the methacrylation reaction. Simultaneously, the methacrylation process of gelatin is typically enhanced by the presence of amine groups in their neutral form (-NH_2_). This occurs when the pH of the solution approaches the pKa value of the epsilon-amino groups found in the lysine residues of gelatin, approximately ranging between 9 and 10. It is important to note that manually adjusting the pH can be inconsistent, and the degree of substitution (DOS) heavily relies on precision. Therefore, employing a 0.25 M CB buffer, pH = 9.0, is recommended, as it reduces labor while ensuring that the pH remains above 7 throughout the reaction.

The synthesis of GelMA from gelatin and MAA was confirmed by HNMR spectra, as shown in Figure 3a. In gelatin, a peak at ~2.9 ppm commonly corresponds to the hydrogen atoms in the methylene (-CH_2_-) groups from lysine units [45]. When gelatin was modified by the introduction of methacrylate groups through esterification with methacrylic anhydride, the local chemical environment or electronic environment of these hydrogen atoms changed, represented by a shift or merger with other peaks, resulting in the disappearance of the peak around ~2.9 ppm in GelMA samples. Compared to unmodified gelatin, the appearance of a more pronounced peak at ~1.8 ppm in GelMA was attributed to the methyl protons (-CH_3_) of the grafted methacryloyl group [46]. The peak observed between 5.2 and 6.1 ppm in the GelMA spectra signified the presence of acrylic protons that are attached to the carbon atoms adjacent to the carbon-carbon double bond in the methacryloyl group (-CH_2_-C(CH_3_)=CH_2_) [28]. Conversely, the HNMR spectrum of gelatin predominantly reflected the chemical environment of amino acid residues like glycine, proline, and hydroxyproline [3]. These residues typically lack double bonds or unsaturated functional groups in the gelatin molecule, resulting in the absence of peaks within the 5.2–6.1 ppm range, typically representing hydrogen atoms within double bonds. Collectively, these HNMR spectra are evidence of our successful methacrylation of gelatin.

The ratio of methacrylic anhydride (MAA) to gelatin in GelMA synthesis is an important parameter that influences the degree of methacrylation. The TNBS assay is commonly utilized to quantify the residual free amino groups in GelMA, serving as an indicator of the degree of methacrylation. While modification of gelatin with MAA can result in the formation of both methacrylamide and methacrylate groups through reactions with amino and hydroxyl groups, methacrylate groups represent less than 10% of all methacryloyl substitutions, suggesting that amino groups predominantly contribute to the formation of methacrylamide groups in GelMA [47]. Consequently, the TNBS assay provides a substantial reflection of the degree of substitution (DOS) in GelMA. While increasing the MAA/gelatin ratio may initially produce a higher DOS, there are practical limitations beyond which further increases do not significantly enhance the methacrylation efficiency. As shown in Figure 3c, upon reaching a MAA-to-gelatin ratio of 0.4, the DOS exhibited no significant increase with further ratio increments, suggesting a potential saturation point during the methacrylation process under these reaction conditions. Once reached, further MAA addition fails to proportionally enhance the DOS, as all available amino groups have undergone reactions.

The crosslinking of the GelMA macromer occurs when GelMA is exposed to UV radiation in the presence of photoinitiators. Photoinitiators convert this light energy at 365 nm into chemical energy in the form of free radicals and reactive cations, which subsequently initiate the polymerization of GelMA [48]. Irgacure 2959 and LAP have been widely used in the polymerization of GelMA hydrogels [49]. LAP and Irgacure 2959 are categorized as type I photoinitiators that undergo unimolecular bond cleavage upon irradiation to yield free radicals. However, their distinct chemical structures influence the absorption range and quantum efficiency of the photochemical and photophysical processes occurring in excited states [50]. When irradiated, Irgacure 2959 yields two primary radicals, benzoyl and alkyl, whereas LAP generates phenyl and phosphinoyl radicals. The combined presence of these functional groups enables LAP to absorb UV light more effectively than Irgacure 2959, initiating photochemical reactions that drive polymerization in UV-curable materials. Additionally, LAP exhibits greater water solubility compared to Irgacure 2959, potentially resulting in more homogeneous solutions and improved distribution of photoinitiators throughout the material, thereby further enhancing its efficacy [50].

In addition to photoinitiator type, the gelation process of GelMA hydrogels is primarily influenced by the concentration of GelMA macromer, the concentration of the photoinitiator, and the duration of UV exposure, assuming that other variables, such as temperature, UV light intensity, and the degree of methacrylation of gelatin, are constant. As the GelMA concentration increases, more polymer chains are available, resulting in increased opportunities for crosslinking to occur between the methacrylate groups. Longer exposure time during UV curing provides more opportunities for UV light to penetrate deeper into the hydrogel matrix. Deeper penetration ensures that more of the material is exposed to the activating radiation, reaching regions that might not be accessible during shorter exposure times [51]. When the concentration of the photoinitiator increases, additional molecules are available to absorb light energy and generate free radicals or reactive species, leading to the accelerated initiation of the photopolymerization process by reacting with the methacrylate groups on GelMA molecules [52].

The gelation degree of GelMA hydrogels largely dictates their mechanical properties. As the gelation degree increases, the hydrogel becomes stiffer and stronger due to the increased formation of a three-dimensional network of polymer chains. The increased crosslinking density results in a hydrogel that is more resistant to deformation and mechanical stress. Generally, elevating the concentration of LAP, prolonging the duration of UV exposure, and increasing the GelMA precursor concentration enhance stiffness in GelMA hydrogels. However, this pattern was not consistent. As illustrated in Figure 4a,b, although an increasing trend was observed, the change in the compressive modulus was not statistically significant. This outcome stems from the fact that once the crosslinking reaction is complete, an additional LAP concentration or prolonged UV exposure does not induce further crosslinking or significantly alter the network structure. Nonetheless, increasing the concentration of GelMA could produce a greater degree of crosslinking (Figure 4c) due to the presence of more methacrylate groups available for crosslinking reactions. This can lead to the formation of a denser and more tightly crosslinked hydrogel network, resulting in improved mechanical properties and stability.

The stiffness of GelMA hydrogels can be indirectly reflected by SEM images through observation of structural features and morphology. The findings shown in Figure 5a–c aligned with the stiffness trends described in Figure 4. Stiffer GelMA hydrogels tend to exhibit a greater density and alignment of polymer fibers, resulting in smaller and more uniformly distributed pores. In contrast, softer hydrogels may display larger and more irregularly shaped pores due to their lower crosslinking density.

The pore size of GelMA hydrogels can influence the swelling degree by affecting the accessibility of water molecules to the internal structure of the hydrogel. GelMA hydrogels with larger pore sizes generally have more open and interconnected pore structures. These larger pores provide easier access for water molecules to penetrate the hydrogel matrix, leading to greater swelling. Conversely, GelMA hydrogels with smaller pore sizes have more restricted and less interconnected pore structures. The smaller pores limit the diffusion of water molecules into the hydrogel matrix, reducing the accessibility of water to the polymer chains. As demonstrated in Figure 5d, as the GelMA concentration decreased, the pore sizes increased, allowing for greater water uptake and resulting in more extensive swelling of the hydrogel. In contrast, hydrogels with smaller pore sizes exhibited lower swelling degrees than did those with larger pore sizes.

UV light can damage cells primarily through the generation of reactive oxygen species (ROS), leading to oxidative stress and the formation of DNA lesions such as oxidative base modifications and DNA strand breaks [53]. While LAP is considered biocompatible and nontoxic at low concentrations, free radicals generated during LAP photolysis can cause indirect DNA damage within cells [54]. The combination of LAP and UV light may enhance the penetration of ROS into cells, amplifying their cytotoxic effects. This increased ROS production can overwhelm cellular antioxidant defenses, leading to oxidative damage and cell death. Hence, according to the results in Figure 6c, a 0.025% concentration and a 2 min UV exposure were optimal for the study of cell viability within the hydrogels, ensuring complete crosslinking of all samples while minimizing cellular damage.

By examining the fluorescence images, the relative proportion of live and dead cells within the hydrogels was assessed. As shown in Figure 7a, an increase in the GelMA concentration corresponded to an increase in the red fluorescence signal, indicating a decrease in cell viability. This phenomenon arises from the higher concentration of GelMA, which results in the formation of more numerous covalent bonds. Consequently, the hydrogel exhibited increased rigidity and reduced porosity, thereby markedly diminishing cell viability [48].

In addition to live/dead cell viability measurements, the alamarBlue assay was also used in evaluating cell viability. The alamarBlue assay allowed for quantitative assessment of cell viability by measuring the reduction of resazurin dye to its fluorescent product, i.e., resorufin, by metabolically active cells [55]. Metabolic activity is a metric often used as a representation of the overall health and functionality of cells, including their ability to proliferate and maintain homeostasis. This assay provides complementary information to live/dead staining by assessing cellular function rather than membrane integrity alone. The extent of the reduction of alamarBlue is directly proportional to the metabolic activity of the cells. As the reduction of alamarBlue decreased, cell viability also decreased. As shown in Figure 7b, a lower concentration of GelMA was associated with a greater reduction of alamarBlue, indicating that a lower concentration of GelMA increased cell viability. These findings corresponded with the evaluation of cell viability obtained through direct fluorescence staining.

Hematoxylin and eosin (H&E) staining is a commonly used histological staining method that provides insight into tissue morphology and cellular structure. While H&E staining is not typically used to directly assess cell viability in hydrogels, it can indirectly reflect aspects of cell behavior and viability through the visualization of cell distribution, morphology, and organization within the hydrogel matrix. As shown in Figure 7c, the rounded shape of the cells was caused by material on all sides, unlike in traditional 2D culture, where cells adhere to a flat surface. This confinement can restrict cell spreading and the extension of cellular processes, resulting in a rounded morphology. A greater number of viable cells were found in hydrogels with lower GelMA concentrations (7.5% and 10%) than in hydrogels with higher GelMA concentrations (15% and 20%). This is due to the more permeable, less rigid constitution of hydrogels prepared with lower GelMA concentrations, which are less likely to exert mechanical stress on encapsulated cells. Additionally, the reduced density of crosslinks in these hydrogels promotes enhanced diffusion of nutrients and waste products, further supporting cell viability.

## 5. Conclusions

The synthesis of GelMA involves controlling various parameters to tailor its properties for specific applications in tissue engineering and regenerative medicine. This study extensively investigated a straightforward GelMA synthesis method, employing different buffer systems, various ratios of MAA to gelatin type B, and diverse crosslinking conditions. The findings indicated that a simplified synthesis process utilizing a feed ratio of MAA/gelatin of 0.4 mL/g in 0.25 M CB buffer (pH 9) yielded GelMA with a high degree of methacrylation. LAP demonstrated superior efficacy compared to Irgacure 2959 as PI in crosslinking GelMA. GelMA gelation was also influenced by GelMA concentration, UV exposure time, and photoinitiator concentration. The gelation degree was directly related to the mechanical and physical properties of the material, which were significantly impacted by the GelMA concentration. The viability of the TC28a2 chondrocytes used in this study was significantly impacted by the crosslinking conditions. Higher concentrations of GelMA resulted in a firmer hydrogel, leading to reduced cell viability. Although the cytotoxicity increased with increasing LAP concentration and longer UV exposure time, their combination remained biocompatible when the UV exposure duration was limited to 2 min and the LAP concentration did not exceed 0.025%. To ensure complete crosslinking of GelMA while minimizing cell damage, the optimal conditions for TC28a2 chondrocytes cultured in hydrogel were determined to be 7.5% GelMA, 0.025% LAP, and 2 min UV exposure at 365 nm. While the GelMA hydrogel under these conditions, which are suitable for immortalized chondrocytes (i.e., TC28a2), may offer promising characteristics, it may not be suitable for primary cells and stem cells. This is because primary cells and stem cells may be more sensitive to environmental cues and require a more biomimetic microenvironment for proper function and differentiation.

These conditions represent a valuable approach for developing tissue-engineered cartilage constructs with TC28a2 chondrocytes in GelMA, where chondrogenic differentiation is induced by the chondrogenic differentiation medium. However, tissue-engineered cartilage constructs lack the complexity and functionality of native cartilage. Engineered constructs may lack the intricate ECM composition and organization found in native cartilage, which consists of collagen fibers, proteoglycans, and other proteins arranged in a specific hierarchical structure. Chondrocytes encapsulated in hydrogels may not fully replicate the heterogeneity and functionality of cells found in native cartilage. These differences can be revealed by histology, immunohistochemistry, biochemical analysis, and mechanical testing.

Despite their inability to fully substitute for native cartilage, tissue-engineered cartilage constructs hold promise for repairing and replacing damaged or degenerated cartilage in patients with conditions such as osteoarthritis or cartilage defects. Additionally, they can be used for screening potential drug candidates or evaluating the efficacy of therapeutic agents for cartilage-related conditions. This represents the next step in our research process.

## Figures and Tables

**Table 1:** Change of pH value during GelMA synthesis.

	0	30 min	60 min	90 min	120 min	150 min	180 min
CB buffer	9.0	7.16	5.67	5.54	5.4	5.25	5.19
CB buffer pH = 9.0	9.0	9.0	9.0	9.0	9.0	9.0	9.0
PBS buffer	7.4	4.07	3.91	3.78	3.7	3.65	3.63
PBS buffer pH = 7.4	7.4	7.4	7.4	7.4	7.4	7.4	7.4

**Table 2:** Gelation evolution of GelMA precursor with photoinitiator LAP and Irgacure 2959 across various conditions.

Photoinitiator Concentration																													
		0.005%				0.01%				0.025%				0.05%				0.10%				0.25%				0.50%			
	UV	GelMA Concentration (%)																											
	(min)	5	10	15	20	5	10	15	20	5	10	15	20	5	10	15	20	5	10	15	20	5	10	15	20	5	10	15	20
LAP	0.5	⍻	⍻	⍻	⍻	⍻	⍻	⍻	⍻	⍻	⍻	⍻	⍻	⍻	⍻	⍻	⍻	⍻	⍻	⍻	⍻	⍻	⍻	⍻	⍻	⍻	⍻	⍻	⍻
	1	⍻	⍻	⍻	⍻	⍻	⍻	✓	✓	✓	✓	✓	✓	✓	✓	✓	✓	✓	✓	✓	✓	✓	✓	✓	✓	✓	✓	✓	✓
	2	⍻	⍻	⍻	⍻	✓	✓	✓	✓	✓	✓	✓	✓	✓	✓	✓	✓	✓	✓	✓	✓	✓	✓	✓	✓	✓	✓	✓	✓
	4	⍻	⍻	⍻	⍻	✓	✓	✓	✓	✓	✓	✓	✓	✓	✓	✓	✓	✓	✓	✓	✓	✓	✓	✓	✓	✓	✓	✓	✓
	6	⍻	⍻	⍻	⍻	✓	✓	✓	✓	✓	✓	✓	✓	✓	✓	✓	✓	✓	✓	✓	✓	✓	✓	✓	✓	✓	✓	✓	✓
	8	⍻	⍻	⍻	⍻	✓	✓	✓	✓	✓	✓	✓	✓	✓	✓	✓	✓	✓	✓	✓	✓	✓	✓	✓	✓	✓	✓	✓	✓
	10	⍻	⍻	⍻	⍻	✓	✓	✓	✓	✓	✓	✓	✓	✓	✓	✓	✓	✓	✓	✓	✓	✓	✓	✓	✓	✓	✓	✓	✓
Irgacure 2959	0.5	✗	✗	✗	✗	✗	✗	✗	✗	✗	✗	✗	✗	✗	✗	✗	✗	✗	✗	✗	✗	✗	✗	✗	✗	✗	✗	✗	✗
	1	✗	✗	✗	✗	✗	✗	✗	✗	✗	✗	✗	✗	✗	✗	✗	✗	✗	✗	✗	✗	✗	✗	✗	✗	✗	✗	✗	✗
	2	✗	✗	✗	✗	✗	✗	✗	✗	✗	✗	✗	✗	✗	✗	✗	✗	✗	✗	✗	✗	✗	✗	✗	✗	⍻	⍻	⍻	⍻
	4	✗	✗	✗	✗	✗	✗	✗	✗	✗	✗	✗	✗	✗	✗	✗	✗	⍻	⍻	⍻	⍻	⍻	⍻	⍻	⍻	✓	✓	✓	✓
	6	✗	✗	✗	✗	✗	✗	✗	✗	✗	✗	⍻	⍻	⍻	⍻	⍻	⍻	⍻	⍻	⍻	⍻	⍻	⍻	⍻	⍻	✓	✓	✓	✓
	8	✗	✗	✗	⍻	✗	✗	⍻	⍻	✗	✗	⍻	⍻	⍻	⍻	⍻	⍻	⍻	⍻	⍻	⍻	⍻	⍻	⍻	⍻	✓	✓	✓	✓
	10	✗	✗	✗	⍻	✗	✗	⍻	⍻	✗	⍻	⍻	⍻	⍻	⍻	⍻	⍻	⍻	⍻	⍻	⍻	⍻	⍻	⍻	⍻	✓	✓	✓	✓

Note: ✓ indicates completely crosslinked GelMA; ⍻ indicates incompletely crosslinked GelMA; ✗ indicates non-crosslinked GelMA.

## Data Availability

The data are available from the corresponding author upon reasonable request.

## Supplementary Materials

The following supporting information can be downloaded at: https://www.mdpi.com/article/10.3390/jfb15030077/s1; Figure S1: Gelation of GelMA precursor across various conditions with (a) photoinitiator LAP and (b) photoinitiator Irgacure 2959; Table S1: Tissue processing steps for paraffin sections; Table S2: H&E staining steps.
