# Supplementary material for: Precision Engineering of Chondrocyte Microenvironments: Investigating the Optimal Reaction Conditions for Type B Gelatin Methacrylate Hydrogel Matrix for TC28a2 Cells
Source: J Funct Biomater. 2024 Mar 20;15(3):77. doi: 10.3390/jfb15030077 (PMC10971347; doi:10.3390/jfb15030077)
Supplement: Supplementary file 1 [file jfb-15-00077-s001.zip › jfb-2905855-supplementary.pdf]

Journal of *Functional Biomaterials*

Supplementary Materials

Precision Engineering of Chondrocyte Microenvironments: Investigating the Optimal Reaction Conditions for Type B Gelatin Methacrylate Hydrogel Matrix for TC28a2 Cells

Qichan Hu [1], Marc A. Torres [1], Hongjun Pan [2], Steven L. Williams [3] and Melanie Ecker [1,*]

[1] Department of Biomedical Engineering, University of North Texas, Denton, TX 76203, USA; qichanhu@my.unt.edu (Q.H.); marctorres@my.unt.edu (M.A.T.)
[2] Department of Chemistry, University of North Texas, Denton, TX 76203, USA; hongjun.pan@unt.edu
[3] Department of Biological Sciences, University of North Texas, Denton, TX 76203, USA; stevenwilliams5@my.unt.edu
* Correspondence: melanie.ecker@unt.edu

Citation: Hu, Q.; Torres, M.A.; Pan, H.; Williams, S.L.; Ecker, M. Precision Engineering of Chondrocyte Microenvironments: Investigating the Optimal Reaction Conditions for Type B Gelatin Methacrylate Hydrogel Matrix for TC28a2 Cells. *J. Funct. Biomater.* **2024**, *15*, 77. https://doi.org/10.3390/jfb15030077

Academic Editor: Zhen Zheng

Received: 21 February 2024
Revised: 15 March 2024
Accepted: 16 March 2024
Published: 20 March 2024

Copyright: © 2024 by the authors. Licensee MDPI, Basel, Switzerland. This article is an open access article distributed under the terms and conditions of the Creative Commons Attribution (CC BY) license (https://creativecommons.org/licenses/by/4.0/).

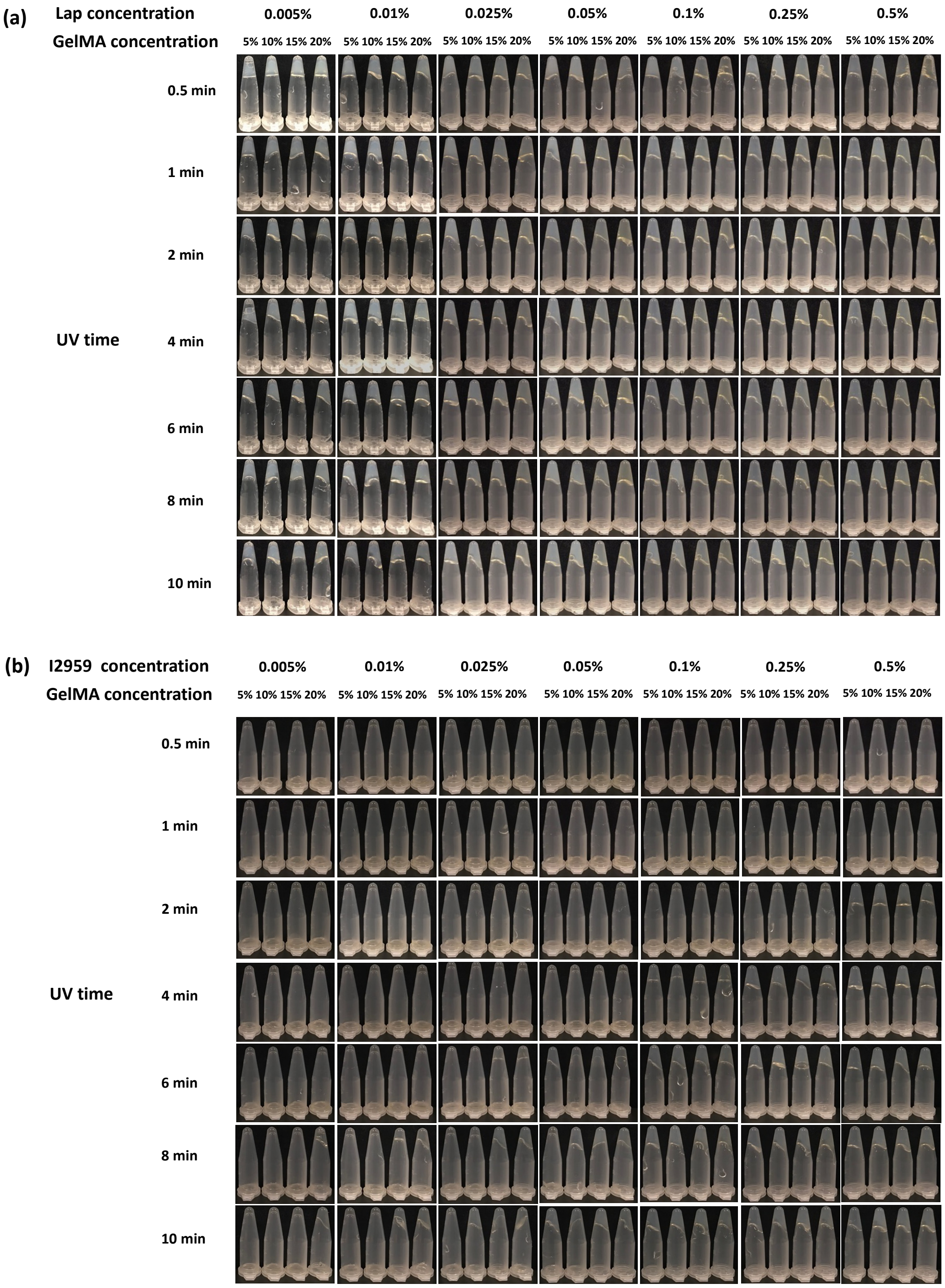

Figure S1. Gelation of GelMA precursor across various conditions with (a) photoinitiator LAP and (b) photoinitiator Irgacure 2959.

Table S1. Tissue processing steps for paraffin sections.

Step	Time (h)	Temperature (°C)
4% PFA Fixation	24	20-22
70% EtOH	1	20-22
80% EtOH	0.75	20-22
95% EtOH	0.75	20-22
95% EtOH	1	20-22
100% EtOH	1	20-22

100% EtOH	1	20-22
100% EtOH	1	20-22
Xylene	1	20-22
Xylene	1	20-22
Xylene	1	20-22
Paraplast®	2	56
Paraplast®	1	56
Paraplast®	1	56

Table S2. H&E staining steps.

Step	Time (min)	Frequency
Xylene	3	3 X
100% EtOH	3	3 X
95% EtOH	3	1 X
80% EtOH	3	1 X
diH_2O	5	1 X
Hematoxylin Solution	3	1 X
diH_2O	2	1 X
Running Tap H2O	5	1 X
diH_2O Rinse	2	1 X
Eosin Solution	2	1 X
95% EtOH	3	3 X
100% EtOH	3	3 X
Xylene	15	3 X

Disclaimer/Publisher's Note: The statements, opinions and data contained in all publications are solely those of the individual author(s) and contributor(s) and not of MDPI and/or the editor(s). MDPI and/or the editor(s) disclaim responsibility for any injury to people or property resulting from any ideas, methods, instructions or products referred to in the content.
